# Mast Cell Proteases Promote Diverse Effects on the Plasminogen Activation System and Wound Healing in A549 Alveolar Epithelial Cells

**DOI:** 10.3390/cells11182916

**Published:** 2022-09-18

**Authors:** Sofia Mogren, Frida Berlin, Lykke Eskilsson, Nicole Van Der Burg, Ellen Tufvesson, Cecilia K. Andersson

**Affiliations:** 1 Department of Experimental Medical Science, BMC, Lund University, 222 42 Lund, Sweden; 2 Department of Clinical Sciences, BMC, Lund University, 222 42 Lund, Sweden

**Keywords:** mast cell, tryptase, chymase, alveolar epithelial cells, wound healing, migration, urokinase plasminogen activator receptor

## Abstract

Tissue damage, epithelial alterations, and intraepithelial presence of mast cells (MCs) are characteristics of asthma pathogenesis. Increased alveolar infiltration of MC populations has also been identified as a feature of asthma and other chronic respiratory diseases. The asthma associated receptor, urokinase plasminogen activator receptor (uPAR), has been shown to regulate bronchial epithelial repair responses. However, the impact of MC tryptase and chymase on functional properties and expression of uPAR in alveolar epithelial cells have not been fully investigated. Alveolar epithelial cell migration and wound healing were investigated using holographic live cell imaging of A549 cells in a wound scratch model post stimulation with tryptase or chymase. The expression of uPAR was investigated on the protein and gene level from cellular supernatants and in bronchoalveolar lavage fluid fractions from allergic asthmatics. We found that tryptase improved wound healing capacity, cellular migration and membrane bound uPAR expression. Chymase reduced gap closure capacity, cellular migration and membrane bound uPAR expression but increased soluble uPAR release. Our data suggest a dual regulatory response from the MC proteases in events related to uPAR expression and wound healing which could be important features in asthmatic disease.

## 1. Introduction

The human mast cell (MC) is an immune cell of hematopoietic origin and like a two-faced joker, the MC is well recognized for its role in pathological settings yet acknowledged for its protective role in health [1]. MCs are abundant in the airway mucosa and epithelium, but also within the septa of the alveolar parenchyma [2]. However, the role of alveolar MCs is largely unknown. MC subpopulations are binary phenotyped by their protease content in granula; MCs storing tryptase (MC_T_) and MCs storing tryptase and chymase (MC_TC_) [1]. In pulmonary baseline conditions MC proteases have a key role in homeostasis and wound healing; enhancing migration, proliferation, and gap closure capacity, but also inducing the release of growth factors associated with tissue repair in fibroblasts and epithelial cells [3,4,5,6]. However, in an asthmatic context an increase of MCs and change of distribution and phenotype in both bronchial and alveolar compartments often associate with disease severity [2,7]. It has also been shown that MC proteases contribute to airway bronchoconstriction, hyperresponsiveness and airway remodelling [8]. 

The binding of urokinase plasminogen activator (uPA) to its glycosylphosphatidylinositol-inositol (GPI) anchored plasminogen activator urokinase receptor (uPAR) initiates several cellular pathways that modulate a variety of functions including homeostasis, inflammation, and wound healing [9,10]. The uPA/uPAR system has an important role in the regulation of cellular mitogenic effects such as migration and proliferation in relation to events associated with normal cellular development and tissue repair. However, dysregulated cellular proliferation and migration contributes to plural disorders with remodelling and fibrotic characteristics [9,11]. 

uPAR is composed as a three domain (D1, D2 and D3) GPI anchored membrane bound receptor, but also exists as an anchored cleaved form referred to as suPAR, with high affinity for the serine uPA. The zymogen form of uPA, pro-uPA, also binds to uPAR but with less affinity [9,12]. The uPA molecule is produced by smooth muscle, endothelial and epithelial cells as wells as fibroblasts and monocytes. It is secreted in its zymogen single chain form and by proteolysis it is actuated into a two-chain active form [9,10,12]. The structure of the uPA molecule is trimeric and divided into amino-terminal “growth factor domain”, the kringle domain and the carboxy-terminal catalytic domain [9]. The binding of uPA-uPAR occurs between uPA’s “growth factor domain” and uPAR’s domain D1 as well as D3 [10]. The most frequent description of uPA-uPAR interaction is the role of regulator of the plasminogen activation system, where uPA converts plasminogen to protease plasmin. Plasmin has a key role in degradation of extra cellular matrix components (ECM), lysis of fibrin clots and activation of matrix metalloproteases (MMPs) [12,13]. The uPA-uPAR interaction generates a positive feedback loop since plasmin can cleave and activate the zymogen to uPA’s active two chain form. The system is regulated negatively by covalent binding and inhibition of proteolytic activity between serine protease inhibitor plasminogen inhibitor 1 (PAI-1) and plasminogen inhibitor 2 (PAI-2) and their targets [10,12]. 

Studies have shown that the u-PA/u-PAR system is activated during asthmatic conditions as well as in other inflammatory settings [7,11,14,15]. Further, it has been identified that MC tryptase is a potent activator of pro-uPA and that MC chymase increases levels of suPAR in fibroblasts [16,17]. 

Asthma is a disorder characterized by remodelling and most asthma studies have focused on the large airways. However, MCs are present but less studied in the peripheral and alveolar compartments of the lung [2,18,19]. The uPA-uPAR system has been found to regulate epithelial repair mechanisms [11]. Given this, it is established that MC proteases and the uPA-uPAR system are implicated in asthma; however, the outcome of their interactions in asthmatic disease has not fully been understood. Therefore, by using human alveolar basal epithelial cells (A549) we were able to examine the effect of MC tryptase and chymase on cellular functional properties and the protease impact of uPAR expression. We hypothesize that MC proteases will have an impact of functional structures such as wound healing, migration, and morphology in alveolar epithelial cells. Further, we also expected an impact of uPA/uPAR expression and uPAR related cellular events such as migration in the presence of MC proteases. Our data can confirm earlier bronchial findings of the role of tryptase in an alveolar context with increased gap closure capacity and enhanced migration. However, an opposite effect was observed in chymase stimulated cells. We can also report that tryptase upregulated the expression of the membrane bound uPAR while chymase increased the release of the soluble (suPAR) receptor. Finally, we identified that dual asthmatic responders had higher concentrations of suPAR compared to single asthmatic responders. However, single asthmatic responders had more suPAR in the alveolar fractions compared with the bronchial fractions. Our findings present the possibility that MC via their proteases may contribute to dual regulation on parameters associated with repair mechanisms that are important features in asthmatic disease. 

## 2. Materials and Methods

### 2.1. Subjects

This study included 15 non-smoking patients with mild to moderate allergic asthma of whom 9 had received a low dose of inhaled corticosteroids and the remainder were steroid naïve. The subjects displayed either early allergic reaction only (termed as single responders) or both early and a late allergic reaction (dual responders) post inhaled allergen. No other significant differences in patient characteristics were found (see Table 1 for details). All subjects gave their written informed consent and the Regional Ethics Review Board in Lund, Sweden, approved the study (2012/800). The subjects have been used in previously published studies [20,21]. Subjects underwent bronchoscopy as previously described [21] before and 24 h post allergen challenge where 3 × 50 mL phosphate-buffered saline (PBS) was instilled via a bronchoscope to the middle lobe. The first 50 mL was collected separately and referred to as bronchial lavage and the remaining PBS was recovered as alveolar fraction. Dual and single responders were determined by a reading of the FEV_1_ between 0–2 h and 4–8 h after an administrated allergen challenge. For the current study, samples collected at baseline were used. 

### 2.2. Cell Culture

Alveolar basal epithelial cells (A549) from ECACC (Porton Down, Salisbury, UK) were cultured in Ham’s F-12 nutrient Mix (Gibco, 21765-037) with 10% fetal bovine serum (FBS) and 1% penicillin-streptomycin from Life Technologies (Stockholm, Sweden). For detachment of cells, trypsin-EDTA 0.25% was used (Gibco, 25200056). For the experimental set-up (RNA, protein and supernatant) cells were cultured in a 6 well Nunc multidish (Nunc Technologies, Carlsbad, CA, USA) at 37 °C in 5% CO_2_ and were grown until approximately 80–90% confluency which was determined automatically using a live cell imaging system (Holomonitor Phase Holographic Imaging, Lund, Sweden). Cells were treated with 0.5 μg/mL human lung tryptase (Merck Millipore, Darmstadt, Germany) and 0.5 μg/mL recombinant chymase (Sigma-Aldrich, St. Louis, MO, USA, C8118-50 μg) [5,6]. During cellular experiments starvation medium was used (Ham’s F-12 nutrient Mix medium with 1% FBS and 1% penicillin-streptomycin). RNA and supernatants were collected at 6 h and protein and supernatant were collected 24 h post stimulation. 

### 2.3. RNA Extraction and RT^2^ PCR Array

After 6 h stimulation, mRNA was collected and isolated with Aurum total RNA MiniKit (Bio-Rad, Hercules, CA, USA, 7326820). RNA concentration was measured with nanodrop 2000c Thermo Scientific (Waltham, MA, USA). Using RT^2^ First Strand Kit from QIAGEN (Hilden, Germany), RNA was transcribed to cDNA. For PCR analysis, RT^2^ SYBR Green Rox Master Mix (QIAGEN, 330521) and RT^2^ primer PLAUR (u-PAR) (PPH00797B) were used. For housekeeping, genes ACTB (QIAGEN, PPH00073G) and HPRT1 (QIAGEN, PPH01018C) were used. 

### 2.4. ELISA and LDH Test

Lung lavage fractions from allergic asthmatics and cellular supernatant from A549 cell line and secretion of protein release were analyzed with ELISA DuoSet Kits from R&D systems (Minneapolis, MN, USA) for soluble uPAR (DY807), MMP-9 (DY911-05), PAI-1 (DY1786) and uPA (DY1310). In order to measure possible cytotoxicity, lactate dehydrogenase (LDH) test was used (Roche Diagnostics, Basal, Switzerland). Supernatant was collected at 1 h, 6 h and 24 h and testing was done according to the manufacturer’s protocol. 

### 2.5. Western Blot

Protein was collected at 24 h and the concentration was quantified using Pierce BCA Protein Assay Kit. Samples were diluted with Laemmeli and boiled at 95 °C for 5 min using QIA Amplifier 96 QIAGEN (Hilden, Germany). Samples were added to Mini Protean TGX stain free gel (Bio-Rad, 4668086) and blotted with Transfer Blot Turbo (Bio-Rad, 1704156). The membranes were incubated with uPAR antibody 1:1000 (Cell Signaling, D4Q5S), and reference gene ß-actin, diluted 1:1000 (Cell signalling, Danvers, MA, USA, D6A8) overnight at 4 °C on a sample rocker. Thereafter, the membranes were incubated with secondary HRP conjugated antibody diluted 1:5000 (Bio-Rad, 5213-2504). For chemiluminescent detection, LI-COR Odyssey Fc and Image analysis was performed with Image Studio ver.5.2 (Lincoln, NE, USA). 

### 2.6. Immunocytochemistry

A549 were cultured in chamber slides (Merck Millipore, Darmstadt, Germany) until 60% confluency or 90% confluency for scratch treatment. Tryptase and chymase were added and samples were fixated in 2% paraformaldehyde for 20 min post 24 h addition. For scratch staining, the samples were wounded with a single cut using 200 μL pipette tip prior to addition of proteases. Thereafter, samples were incubated with 0.1% Tween-20 for 10 min. Samples were immunostained with Primary antibody diluted 1:50, uPAR (Cell Signaling, D7X2N) and thereafter labelled with Alexa Fluor 488 or Alexa Flour 647 diluted 1:200 (Invitrogen, Eugene, OR, USA). For double immunostaining of uPAR in relation to the cytoskeleton arrangement, FITC conjugated phalloidin (Sigma-Aldrich, St Louis, MO, USA) was added and diluted 1:1000 post removal of secondary Alexa Fluor antibody. ProLong Glass antifade with Nucblue^TM^ (Thermo Fisher Scientific, Waltham, MA, USA, P36983) were used for mounting and nuclei visualization. A Nikon eclipse 80i combined with Nikon DS-QI1MC was used to view and document images. 

### 2.7. Live Cell Imaging

For functional studies of wound gap closure and migration, A549 were cultured in a Sarstedt TC 6-well plate (Nümbrecht, Germany). For study of wound gaps, model cells were applied to culture-insert 2 well (Ibidi, Martinsried, Germany) with the concentration of 5 × 10^5^ cells/mL. After 24 h, the inserts were removed, and cells were treated with tryptase or chymase with the working concentration of 0.5 μg/mL. For studying the uPA-uPAR effect, cells were pre-treated with uPA-uPAR inhibitor IPR-803 (Sollentuna, Sweden, HY-111192) at the concentration of 50 μM for three hours at 37 °C in 5% CO_2_ and thereafter washed three times with PBS prior to addition of MC proteases. A cell suspension with tryptase was pre-treated for 30 min at 37 °C with 200 μM of a broad serine protease inhibitor, 4-(2-aminoethyl) benzene sulfonyl fluoride hydrochloride (AEBSF) (Sigma-Aldrich, Stockholm, Sweden) in order to confirm the tryptase effect on a functional level. Cells were monitored for 30 h using HoloMonitor M4 live cell imaging system Phase Holographic Imaging (Lund, Sweden). Three randomly chosen positions in each well were selected for image capture with images taken every 15 min, resulting in a total of 2322 images. Quantitative phase imaging of gap closure was done in Holomonitor App Suite version 3.4.0.158 and quantification is based on the fold change of cell covered area. Analysis of cellular migration was performed as earlier described [5]. Briefly, it used single cell tracking of a minimum of 9 randomly chosen cells per well, and technical repeats from the recorded images were performed. The software track computed the tracking data and calculated the cellular movements. However, the migration is based on data from the last 6 h of imaging, timepoint 24 h to timepoint 30 h. This to avoid issues related to cells migrating out of the picture, which prevents adequate monitoring of cells from start at 0 to endpoint at 30 h. Wound healing and migration data are based on three technical repeats. Migration was measured in µm and refers to the distance between start position and end position. Motility was also measured in µm and refers to the entire distance including spontaneous movements. Cellular speed was measured in µm/hour and morphology refers to the measured optical volume (µm^3^) and thickness (µm) of the cells. 

### 2.8. Confocal Microscopy

The samples were visualized on a Nikon A1+ confocal microscope with a 20× Plan Apo objective (NA 0.75) (Nikon Instruments Inc., Tokyo, Japan). Images were acquired and processed with NIS-elements, version: 4.60.02, (Laboratory Imaging, Nikon, Tokyo, Japan).

### 2.9. Cell Viability

Alveolar epithelial cells were cultured and stimulated with MC proteases as described above. Cells were removed using TrypLe Express (12604013, Gibco, Thermo Fisher Scientific, Waltham, MA, USA) and cell suspensions were diluted 1:1 with trypan blue (15250061, Gibco Thermo Fisher Scientific, Waltham, MA, USA). Thereafter, 10 μL of the cell suspension was added to an automatised cell counter (DeNovix, Celldrop Brightfield Cell Counter, Wilmington, DE, USA) for quantification of cell viability where dead cells display an entry of trypan blue and are stained compared to live cells that remain unstained.

### 2.10. Statistical Analysis

Statistical analysis was performed using GraphPad Prism 9.1.2 (GraphPad Software, La Jolla, CA, USA). Nonparametric Mann–Whitney U tests were used to detect differences between 2 groups. For multiple comparison between more than two groups at different time points two-way ANOVA with Tukey’s multiple comparison test was used. Results were considered significant at *p* ≤ 0.05. The data will be presented as Mean ± SD. 

## 3. Results

### 3.1. Chymase and Tryptase Have Discordant Effect on Alveolar Epithelial Wound Healing Capacity

**Figure 1:**
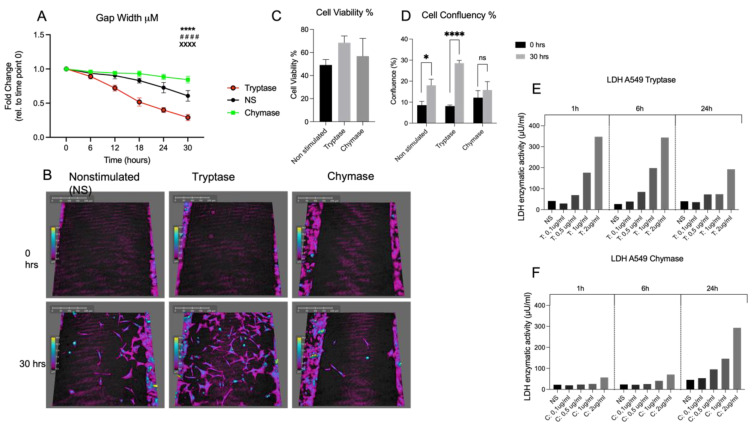
Tryptase enhances gap closure capacity whereas chymase attenuates the gap closure rate compared to nontreated cells in alveolar epithelial cells (**A**). Holographic images of non-stimulated, tryptase and chymase stimulated cells at 0 h and 30 h (**B**). Cell viability (**C**) and cell confluency test (**D**) to further evaluate potential cytotoxic effect of MC proteases. Lactate Dehydrogenase (LDH) cytotoxicity test establishes non-toxic working concentration of tryptase (**E**) and chymase (**F**) in alveolar epithelial cells. Statistical significance between non-stimulated, tryptase and chymase stimulated was tested using a two-way ANOVA with Tukey’s multiple comparison test. Data represent mean (SD), non-stimulated vs. tryptase **** *p* < 0.0001, tryptase vs. chymase xxxx *p* < 0.0001, non-stimulated vs. chymase #### *p* < 0.001. Data based on three technical repeats with three monitored positions in each well. For (**D**), * *p* < 0.05 cell confluency quantified at 0 h and 30 h using holographic live cell imaging **** *p* < 0.0001 cell confluency quantified at 0 h and 30 h post tryptase stimulation using holographic live cell imaging.

Prior to the start of the experiment, lactate dehydrogenase (LDH) was measured, and no cytotoxic potential was identified for the chosen working concentrations of tryptase and chymase (both 0.5 µg/mL) at any of the time points 1 h, 6 h and 24 h (Figure 1E,F). To further evaluate potential cytotoxic/cytostatic effects of MC proteases, cell confluence was quantified at 0 and 30 h, which revealed no reduction of cell confluency in tryptase and chymase stimulated cells 30 h post stimulation (Figure 1C). Cell viability was also investigated and showed no decrease in cell viability in the tryptase or chymase stimulated groups compared to untreated controls (Figure 1D). Monitoring alveolar epithelial cells stimulated with tryptase or chymase for 30 h using a live cell imaging system revealed dual actions of MC proteases on the functional level. We found that tryptase improved alveolar gap closure by decreasing the gap width between wound patches from the starting time point to end time point at 30 h (0.63 ± 0.28 μm *p* < 0.0001) compared to non-treated cells (0.83 ± 0.14 μm) and chymase stimulated cells (0.92 ± 0.05 μm *p* < 0.0001) (Figure 1A,B). In contrast to tryptase, chymase stimulated cells had reduced capacity of wound healing compared to nontreated cells (*p* < 0.0001). For live cell imaging capture of the different treatment groups please see video in supplementary material.

### 3.2. Tryptase Improves and Chymase Impairs Cell Migration, Motility, and Cellular Speed in Alveolar Epithelial Cells

**Figure 2:**
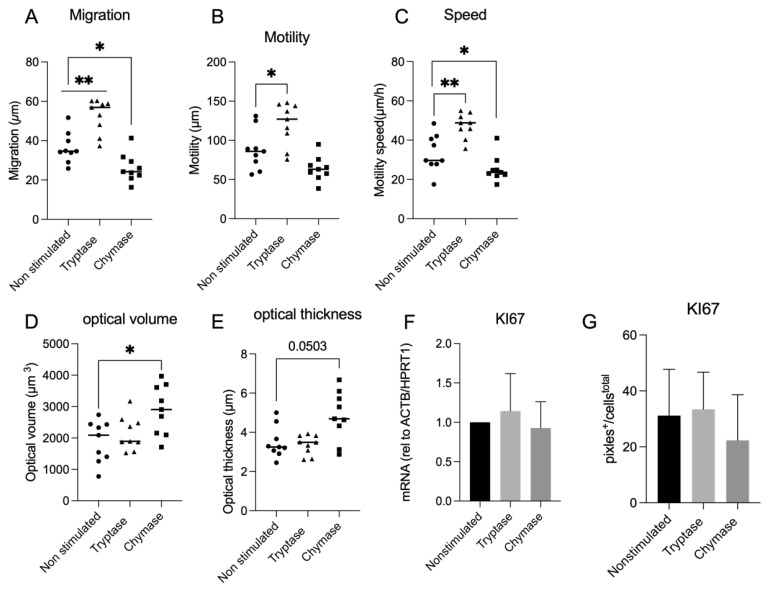
Chymase reduced and tryptase enhanced the migrated distance in µm from start point to end point (linear distance) compared to non-stimulated cells (**A**). Tryptase also enhanced the motility (total migrated distance in µm) compared to both chymase and non-stimulated cells (**B**). Tryptase increased the cellular speed (µm/h) compared to non-stimulated cells and chymase decreased the cellular speed compared to non-stimulated cells (**C**). Chymase stimulated cells displayed altered morphology in terms of optical volume compared to tryptase and non-stimulated cells (**D**) and chymase stimulated cells also displayed a trend of enriched optical thickness compared to both tryptase and non-stimulated cells (**E**). mRNA and protein expression of proliferation marker Ki67 did not display any significant difference between the groups (**F**,**G**). Statistical significance between non-stimulated and tryptase and non-stimulated and chymase stimulated was tested using the Mann–Whitney test. Data represent mean (SD). * *p* < 0.05, ** *p* < 0.01. Data are based on a minimum of three technical repeats.

To investigate cellular migration in a wound gap model, a single cell tracking analysis of 3 randomly chosen cells per focus point (9 cells per well and 27 cells in total) was performed. We compared cellular movement between groups over 6 h at time point 24 h to 30 h. The migration capacity (linear distance: shortest distance from start to end point) was enhanced in tryptase stimulated cells (52.68 µm ± 8.619) compared to non-stimulated (36.39 µm ± 7.77 *p* < 0.01) and for chymase stimulated cells the migration capacity was reduced compared to nontreated cells (26.21 µm ± 7.240, *p* < 0.01) (Figure 2A). We identified similar cellular behavior for motility (the total distance moved) between the groups: non-stimulated cells had a motility of (87.88 µm ± 25.69), tryptase stimulated cells (120.60 µm ± 27.05) and chymase stimulated cells (63.92 µm ± 15.63). However, the difference was only significant between tryptase and non-stimulated cells (*p* < 0.05) (Figure 2B). Regarding the motility speed (µm/h), tryptase displayed an upregulated cellular speed (47.37 µm/h ± 6.33) compared to non-stimulated cells (33.46 µm/h ± 9.44) (*p* < 0.01). Chymase treated cells had decreased cellular speed (25.40 µm/h ± 6.67) compared to nontreated cells (*p* = 0.0424) (Figure 2C). We also obtained morphology data in terms of optical thickness (µm) and optical volume (µm^3^). There was no difference between tryptase and non-stimulated groups ((tryptase: optical thickness (3,35 µm ± 0.49), optical volume (2149 µm^3^ ± 541.40), non-stimulated: optical thickness (3.48 µm ± 0.80), optical volume (1890 µm^3^ ± 664.80)). However, chymase treated cells demonstrated an increase of optical volume compared to nontreated cells (4.829 µm^3^ ± 1.27) (*p* < 0.05) (Figure 2D) and a trend of change in optical thickness compared to nontreated cells (4.82 µm ± 1.27) (*p* = 0.0503) (Figure 2E). We also investigated proliferation, revealed as the expression of the nuclei target Ki67 on mRNA and protein level. However, there was no pronounced proliferative effect of either tryptase (1.387 ± 0.4765) or chymase (1.113 ± 0.2859) compared to untreated cells (Figure 2F,G). In summary, our results indicate that tryptase and chymase have a greater effect on alveolar cell migratory than proliferative properties during wound healing. 

### 3.3. Tryptase Induces Expression of Membrane Bound uPAR Both on Protein and mRNA Levels in Alveolar Epithelial Cells

**Figure 3:**
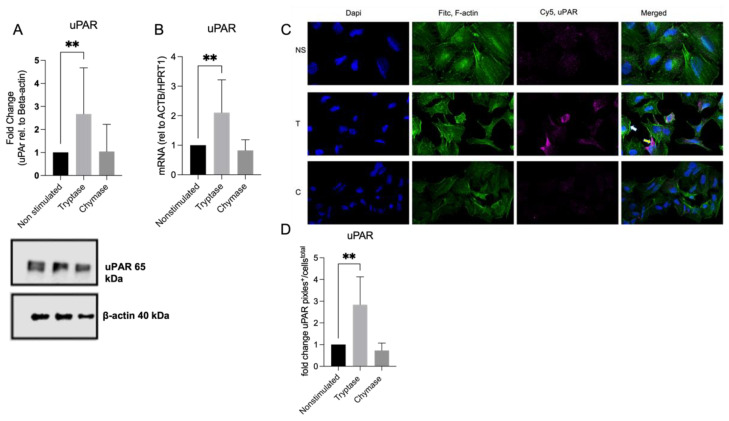
Cell lysates analyzed with Western blot and results based on quantification of fold change. Tryptase stimulated cells augmented the expression of uPAR (65 kDA) on protein level compared to non-stimulated cells (**A**). Tryptase stimulated cells enhanced the expression of uPAR on gene level (**B**). Double staining visualized with confocal microscopy of alveolar epithelial cells targeting F-actin (FITC/green) and uPAR (Cy5/Purple) revealed a stronger signal of uPAR (**C**,**D**) and a preference of a localization in a migratory direction (not quantified) in tryptase stimulated cells (yellow arrow) compared to non-stimulated cells. Statistical significance between non-stimulated and tryptase stimulated cell was tested using the Mann–Whitney test. ** *p* < 0.01. Data represent mean (SD) and a minimum of three technical repeats was used for quantified data.

Exploring the MC protease impact of the membrane bound uPAR receptor revealed that tryptase enhances the expression of uPAR both on the protein and gene level (Figure 3A–D). Protein analysis of Western blot gels showed that tryptase stimulated cells had augmented expression of the membrane bound uPAR receptor (65 kDa) compared to nonstimulated cells (fold change: 2.10 ± 1.10, *p* < 0.01) (Figure 3A). Chymase stimulated cells did not display any significant change of uPAR expression compared to non-stimulated cells (fold change: 0.82 ± 0.36, *p* = 0.6825) (Figure 3A) on protein level. Using confocal microscopy and immunofluorescence targeting F-actin (Fitc, green) and uPAR (Cy5, purple), we aimed to investigate the expression of uPAR in relation to cytoskeleton composition. Again, we could confirm an enhanced protein expression of uPAR in the tryptase stimulated cells compared to the controls (*p* < 0.01) (Figure 3C,D). We could also detect a tendency of increased localization of uPAR expression in cells showing a migratory phenotype (e.g., clusters of F-actin positivity in lamellipodia) in the tryptase stimulated cells compared to non-treated and chymase stimulated cells that displayed a more even distribution of uPAR and less clustering of F-actin filaments (Figure 3C); however, we were not able to quantify this observation in an adequate manner. Further, we investigated the uPAR expression on the gene level, where tryptase (fold change: 2.10 ± 1.10 *p* < 0.01), but not chymase (fold change: 0.82 ± 0.36), stimulated cells displayed an upregulated expression of uPAR compared to nonstimulated cells (Figure 3B). 

### 3.4. Chymase Increases the Expression of Soluble uPAR in Alveolar Cells Whereby More Soluble uPAR Is Increased in the Alveolar Fraction from Asthmatic Patients

**Figure 4:**
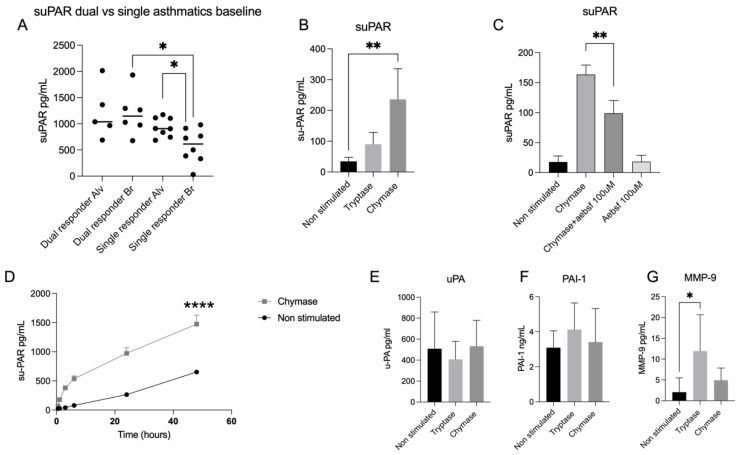
ELISA analysis revealed an increased presence of suPAR in dual responders compared to single responders and higher levels of suPAR in alveolar lavage fractions compared to bronchial BAL in single asthmatic responders (**A**). Chymase stimulated alveolar epithelial cells had increased levels of suPAR compared to non-stimulated cells (**B**) and the chymase effect was confirmed using AEBSF serine protease inhibitor (**C**). The increased liberation of suPAR remained over time (48 h) in chymase stimulated cells compared to non-stimulated cells (**D**). There was no statistical difference between any of the groups regarding the supernatant content of uPA (**E**) or PAI-1 (**F**). Tryptase stimulated cells had a higher protein expression of MMP-9 compared to non-stimulated cells (**G**). Statistical significance between non-stimulated and chymase stimulated cells at different time points was tested using two-way ANOVA with Šidák’s multiple comparison test. Statistical significance between non-stimulated and tryptase stimulated cells and nonstimulated and chymase stimulated cells was tested using the Mann–Whitney test. Data represent the mean (SD) and are based on 5 technical repeats. * *p* < 0.05, ** *p* < 0.01, **** *p* < 0.0001. For dual responders alveolar fraction *n* = 5, dual responders bronchial fraction *n* = 6 and single responders alveolar and bronchial fractions *n* = 8. Br = Bronchial fraction. Alv = Alveolar fraction.

Lung lavage fractions from allergic asthmatic patients were investigated for suPAR using ELISA. Single responders had more suPAR in the alveolar fractions (933.9 pg/mL ± 180.0) compared with the bronchial fraction (578.9 pg/mL ± 323.7) (*p* < 0.05) (Figure 4A). There was also a statistically significant difference between dual and single responders where dual asthmatic responders had more suPAR (1196 pg/mL ± 424.6) compared to single responders (578.9 pg/mL ± 323.7) in the bronchial lavage fractions (*p* < 0.01) (Figure 4A). Analysis of extracellular release from A549 cell line supernatant showed that chymase stimulated cells generated an increased release of suPAR (235 ± 99.52 pg/mL, *p* < 0.01) compared to non-treated controls (34.64 pg/mL ± 12.84) and tryptase stimulated cells (90.20 pg/mL ± 38.75) (Figure 4B). The chymase induced effect on suPAR was also confirmed using a broad serine protease inhibitor AEBSF (Figure 4C). Sampling from different time points up to 48 h showed that the release of suPAR increased over time in chymase stimulated cells compared to non-stimulated cells (Figure 4D). Supernatant from timepoint 0.5 h did not contain statistically significant increased suPAR in chymase stimulated cells compared to untreated controls (*p* = 0.9293). However, from timepoint 1 h there was an increase of suPAR release compared to untreated cells (*p* < 0.01) and, from timepoint 3 h to 48 h, the release of suPAR increased with time (*p* < 0.001) compared to non-stimulated cells (Figure 4D). Further, we measured uPA, a serine protease and the receptor ligand for uPAR. We could not identify any statistically significant different between the groups; however, there was a tendency towards decreased levels of uPA in the tryptase stimulated cells compared to the other groups (non-stimulated: 509.2 pg/mL ± 349.7, tryptase: 407.1 pg/mL ± 172.2 and chymase: 532.7 pg/mL ± 247.6) (Figure 4E). Measurement of the levels of the serine protease inhibitor PAI-1 did not establish any statisically significant difference between the groups, although marginally higher value could be observed in the tryptase stimulated group (4.12 ng/mL ± 0.96) compared to non-stimulated (3.09 ng/mL ± 1.51) and chymase stimulated cells (3.41 ng/mL ± 1.91) (Figure 4F). Finally, we investigated the extracellular liberation of MMP-9 and found that tryptase stimulated cells induced the release of MMP-9 compared to non-stimulated cells (*p* < 0.01) (Figure 4G). Tryptase had a release of 13.66 pg/mL ± 4.82, compared to non-stimulated cells (2.40 pg/mL ± 2.74) and chymase stimulated cells (4.42 pg/mL ± 3.25). 

### 3.5. Inhibition of the uPA-uPAR System Attenuates Gap Closure and Migration of Alveolar Epithelial Cells 

**Figure 5:**
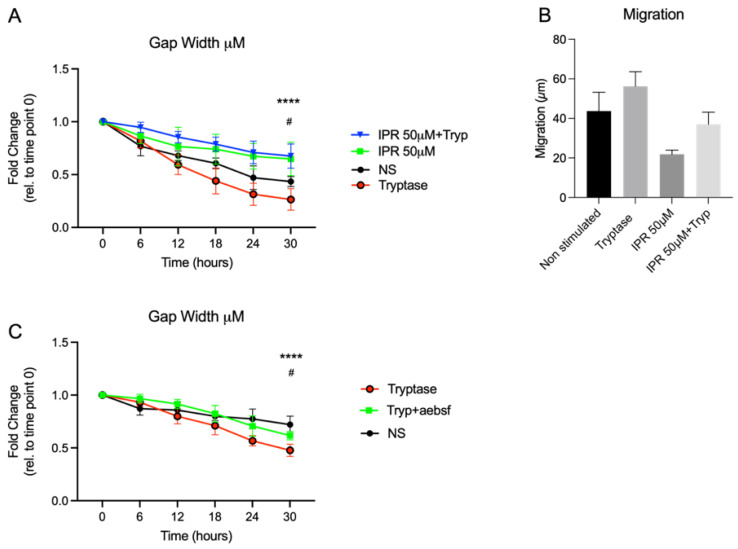
Live cell imaging revealed impaired gap closure (**A**) and migration (**B**) with the addition of uPA-uPAR inhibitor compared to non treated cells. The gap closure promoting effect of tryptase was reduced with the use of serine protease inhibitor AEBSF (**C**). Statistical significance between non-stimulated and chymase stimulated cells at different time points was tested using two-way ANOVA with Šidák’s multiple comparison test. Statistical significance between non-stimulated and tryptase stimulated cells and nonstimulated and chymase stimulated cells was tested using the Mann–Whitney test. Data represent mean (SD) and are based on 3 positions in one technical repeat. Nonstimulated vs. IPR # *p* < 0.05 tryptase vs. tryptase IPR + tryptase **** *p* < 0.0001. Nonstimulated vs. Tryptase # *p* < 0.05, tryptase vs. AEBSF + Tryptase **** *p* < 0.0001.

Functional studies of alveolar epithelial cells revealed a crucial role of the uPA-uPAR system since inhibition of uPA-uPAR promoted impaired gap closure compared to nonstimulated cells (*p* < 0.05) (Figure 5A). Although not statistically significant, alveolar epithelial cells displayed a trend of reduced migration capacity when pre-treated with uPA-uPAR antagonist IPR (non-stimulated: 43.67 μm ± 9.58, IPR: 21.87μm ± 2.10) (Figure 5B). In addition, the pro-migratory effect of tryptase displayed a trend of reduced migration with the addition of IPR (Figure 5B). Further, we confirmed the tryptase effect with improved gap closure activity in alveolar epithelial cells by pre-incubation of tryptase stimulated medium with serine protease inhibitor AEBSF, which reduced the effect of tryptase on the functional level (*p* < 0.05) (Figure 5C). 

## 4. Discussion

Our study revealed that MC tryptase and chymase have binary impacts on alveolar epithelial gap reduction, migration, and morphology. Further, this study demonstrated a dual impact of tryptase and chymase related to the uPAR expression. Current findings could confirm the mitogenic effect of tryptase in terms of gap closure, migration, motility and cellular speed in an alveolar setting that we and others have previously found in human bronchial epithelial cells and fibroblasts [5,6,22,23]. Finally, we can also report an increased released of suPAR from an alveolar lavage fraction compared to bronchial lavage fraction in asthmatic patients with early allergic onset. We also detected a higher release of suPAR in asthmatic patients with both early and late reactions compared with patients with early onset only. 

We could also confirm morphological alterations as an effect of chymase which has previously been shown in fibroblasts [17]. Observed morphological changes in the present study was revealed as a quantitative increase of optical volume and optical thickness. This could possibly be an important observation from the perspective of airway remodelling. Furthermore, tryptase stimulated an increase of MMP-9, which is a metalloproteinase that is strongly related to impacts on the composition of the extra cellular matrix (ECM) [18]. Apart from increase of MMP-9, tryptase stimulated cells also displayed a prominent pro-migratory effect; both are important features of normal physiological development and re-epithelialization upon injury but can also contribute to structural epithelial changes, which are observed in asthma [24,25]. In contrast, our data showed that chymase had an impaired effect on gap closure, migration, motility, and cellular speed. Our data highlight the necessity for a detailed understanding of the influence of tryptase and chymase on a molecular level and how the interaction with epithelial cells might differ depending on the micro localization. This finding may have clinical implications since chymase positive MCs increase in the alveolar compartment in several chronic respiratory diseases such as uncontrolled asthma, COPD and IPF [2,26] where parenchymal remodelling is an important feature. 

The uPA-uPAR system participates in normal cellular development as well as in tissue remodelling with plural downstream effects related to ECM degradation but also changes in functional properties such as migration or proliferation. Accumulating evidence suggests a pivotal role for the uPA-uPAR system in chronic respiratory diseases, where it may contribute to epithelial alterations that generate airway remodelling [11,12,27]. Since the accumulation of activated MCs is an established hallmark of asthma, we wanted to investigate the expression of uPAR in alveolar epithelial cells in the presence of MC proteases tryptase and chymase. Our findings reveal that chymase increases the release of suPAR in alveolar epithelial cells. This has previously been shown in fibroblasts [17] but to the best of our knowledge it has not been found in an alveolar epithelial context before. We can also report that the effect of chymase remained over time (48 h), suggesting not only an enzymatic effect of chymase activity, that has shown to decrease rapidly within the first 6 h of stimulation [5], but also the involvement of possible other mechanisms. Considering that increased levels of suPAR are connected with asthma prevalence [27] and that increased alveolar infiltration of MC_TC_ is a fingerprint of asthmatic disease severity [2], the chymase effect of suPAR is a highly interesting target for future validation in asthmatic disease. We can in the current study report an increase of suPAR levels in the alveolar lavage fractions compared with the bronchial lavage fractions in allergic asthmatics. Further, we could also detect higher levels of suPAR in dual responders compared with single responders, which indicates that suPAR could be correlated with asthma severity since dual responders display a vast and long-lasting airway inflammation compared to single responders [20]. Another finding in our study showed that tryptase stimulated cells had higher expression of uPAR both on protein and gene level. Increased levels of uPAR have been shown to contribute to impaired wound healing capacity in asthmatic bronchial epithelium [11]. We cannot explain why elevated levels of uPAR in our model are associated with improved wound healing. However, since previous studies were conducted on primary bronchial epithelial cells from asthma patients, it is likely that functional and immunological properties may have differed from the commercial cell line of alveolar origin that was used in the current study. It is also important to keep in mind that improved gap closure in vitro could cause events related to tissue remodelling and altered wound healing in an in vivo setting. 

Regarding the serine protease ligand uPA, we could detect a decrease of uPA, but not significant, in the tryptase stimulated cells compared to both chymase- and non-stimulated cells. Since it has been shown that tryptase via proteolytic activity is a potent activator of the zymogen form of uPA and that blocking of the uPA-uPAR system attenuates tissue repair [11,16], this could be a possible explanation for the decreased levels of uPA and the enhanced gap closure activity in our investigation. Given the fact that tryptase just like uPA is a serine protease, this makes tryptase an interesting target for future investigation of its role and interaction with the uPA-uPAR system. 

In our study, tryptase and chymase displayed distinct effects on cellular function but also on expression patterns of mediators of the plasminogen activation system. Since tryptase and chymase might be simultaneously released by certain MC phenotypes, one can speculate on an enhanced dual effect: tryptase induces increased expression of the membrane bound receptor uPAR while chymase cleaves it to its soluble form, suPAR. Previous studies have found increased uPAR expression in the bronchial epithelium of patients with asthma. The study also showed that cells with elevated levels of uPAR showed diminished repair via sequestration of uPA by soluble uPAR [11]. Since the tryptase and chymase expressing MC phenotype, MC_TC_, increases in patients with chronic respiratory disease, including in the alveolar parenchyma in patients with asthma [2], it might indicate a detrimental role for MC_TC_ with regard to uPAR/suPAR expression and release. In depth studies of this pathway in primary cells and in vivo settings is, therefore, needed to fully understand the potential role of MC_TC_ in this context. 

It is important to understand the results in our study from the perspective of MC plasticity, where both cellular origin and in vitro environment most likely have great influence on protease activity and cellular effects. The protease working concentration in our study was set at 0.5µg/mL for both tryptase and chymase. These doses were established since they were non-toxic, while yet displaying an impact on alveolar functional properties. Nevertheless, it should be emphasized that it is difficult to establish a concentration that would be representative for in vivo release considering that MCs contain a high content of bioactive products with a powerful impact on the surrounding environment [28]. The A549 cell line we worked with in the current study has been used earlier to study MC interaction as well as remodelling in an asthma context [29,30]. However, the true and complete nature of the human MC and its proteases in relation to alveolar epithelial cells remains unanswered; hence, future investigations, in clinically relevant settings, are warranted. 

## 5. Conclusions

In conclusion, the current study provides further insight into the impact of MC proteases on alveolar epithelial gap closure capacity, migration, and morphology, where tryptase and chymase induced opposite effects. We could also report a binary impact on the uPAR receptor in the presence of tryptase or chymase, where tryptase upregulated the expression of the membrane bound receptor and chymase increased the release of the soluble receptor. Our in vivo data from asthmatic subjects suggest that infiltration of chymase positive MCs leads to an increase of suPAR that may have clinical implications. Our data suggest a dual regulatory response from the MC proteases in events related to uPA-uPAR expression, ECM degradation and barrier function that may have important roles in the pathogenesis of asthmatic disease. 

## Figures and Tables

**Table 1:** Subject Characteristics.

	Dual Responders	Single Responders	*p*-Value
Subjects, *n*	7	8	
Gender, men/women	3/4	3/5	
Age, years	25 (20–39)	27 (22–45)	
BMI ICS (μg) Asthma duration (years) ACT	24.44 (20.52–37.98) 200 (0–400) 17 (12–39) 21 (18–24)	23.40 (20.20–26.59) 0 (0–400) 19.50 (10–43) 22 (21–25)	ns ns ns
Lung function,			
PD_20_ FEV_1_	222.3 (58.32–555.4) 3.52 (3.27–4.08)	134.5 (64.33–445.9) 3.865 (2.850–4.610)	ns ns
FEV_1_ %predicted	94.80 (84.0–111.7)	98.50 (82.10–119.8)	ns

FEV_1_ = forced expiratory volume in 1 s. Data is presented as median (range). Nonparametric Mann–Whitney test was used to determine statistical difference (*p* < 0.05 considered statistically significant).

## Data Availability

The datasets analyzed during current study will be available from the corresponding author on reasonable request.

## Supplementary Materials

The following supporting information can be downloaded at: https://www.mdpi.com/article/10.3390/cells11182916/s1 Video S1: Live cell imaging of nonstimulated, tryptase and chymase stimulated cells.
