# Supplementary figures and images for: Mast Cell Proteases Promote Diverse Effects on the Plasminogen Activation System and Wound Healing in A549 Alveolar Epithelial Cells
Source: Cells. 2022 Sep 18;11(18):2916. doi: 10.3390/cells11182916 (PMC9496743; doi:10.3390/cells11182916)

## Slide 1
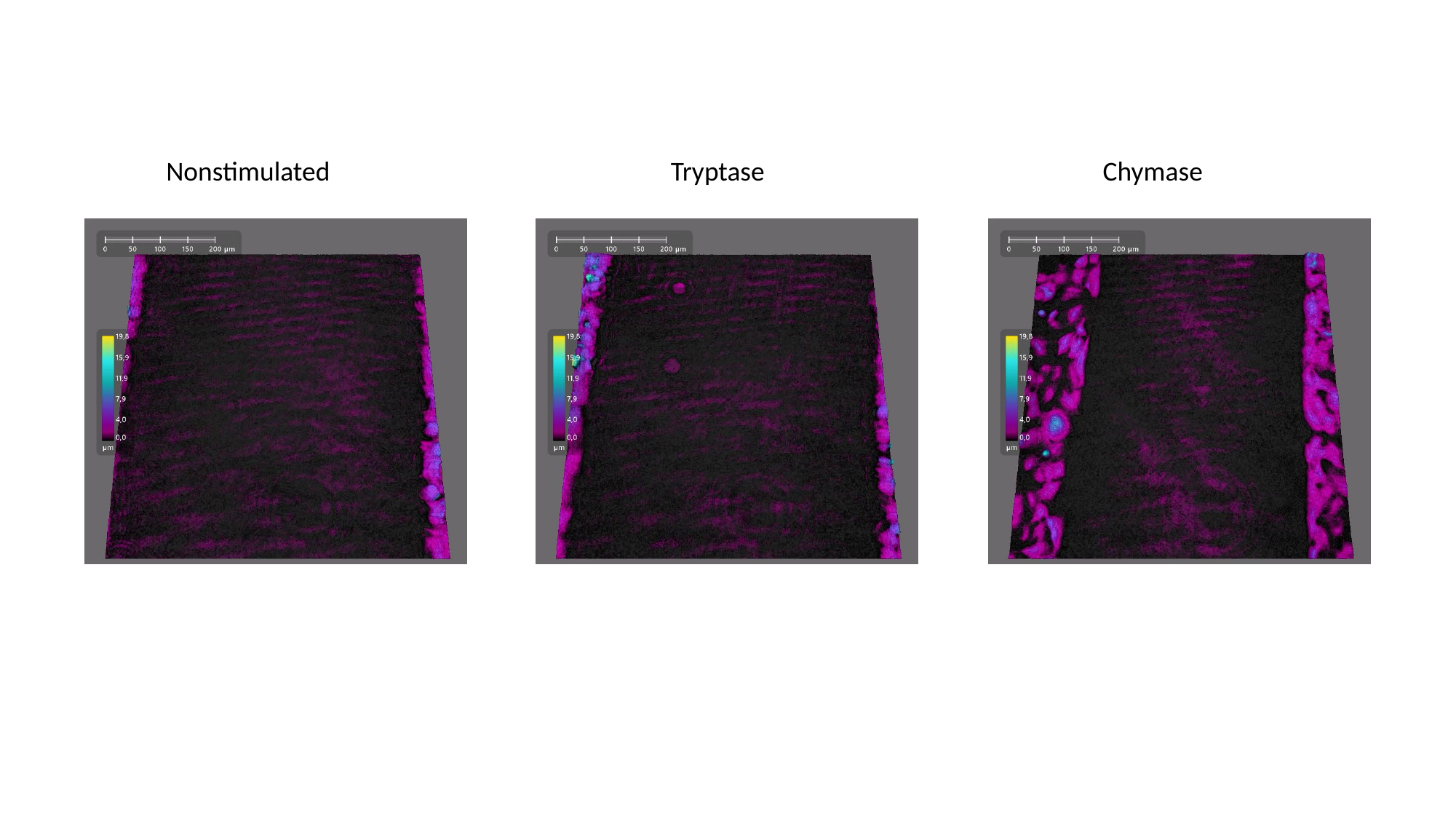

Nonstimulated
Tryptase
Chymase

Supplement: Supplementary file 1 [file cells-11-02916-s001.zip › cells-1929754-supplementary.pptx]
